# P-72. The Evaluation of Pressure Injury Associated Pelvic Osteomyelitis in Patients with Spinal Cord Injuries in a Veterans Affairs Population

**DOI:** 10.1093/ofid/ofae631.279

**Published:** 2025-01-29

**Authors:** Esther H Kanner, Jaclyn A Cusumano, Swapna Johnson-Kunjukutty, Olga Sherman, Kirsten Vest

**Affiliations:** Long Island Jewish Medical Center/North Shore University Hospital; Northwell Health, Brooklyn, New York; Long Island University, Brooklyn, New York; James J. Peters VA Medical Center, New York Medical College, Bronx, New York; James J. Peters VA Medical Center, Bronx, New York; James J. Peters VA Medical Center, Bronx, New York

## Abstract

**Background:**

Limited data, and thus no clinical guidelines, are available for the treatment of pelvic osteomyelitis with associated pressure injuries (PI) in spinal cord injury (SCI) patients. Treatment of SCI patients with PI-associated osteomyelitis at the Veterans Affairs (VA) involves a multidisciplinary team including SCI, infectious disease, wound care, and surgery specialists. Herein we describe the management and outcomes of an SCI cohort with PI-associated pelvic osteomyelitis.

**Figure:**
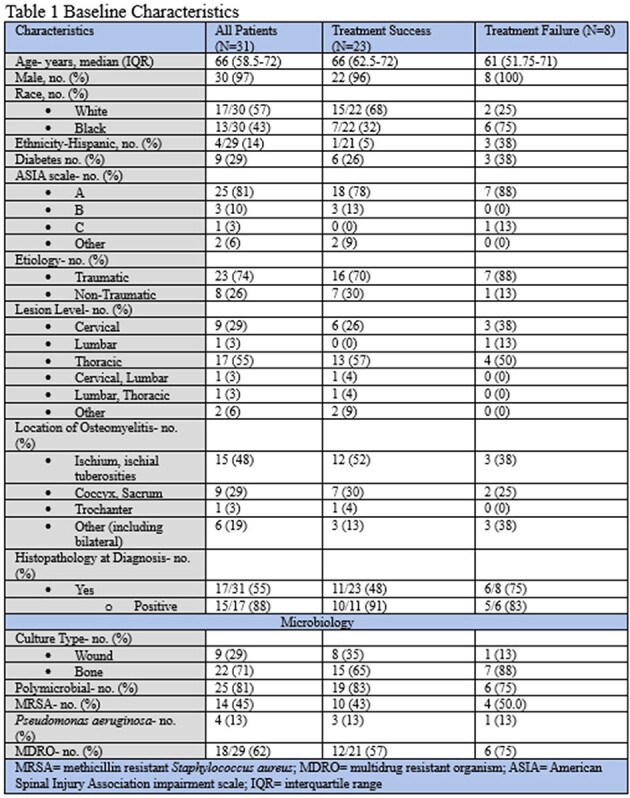

**Methods:**

A retrospective chart review of patients ≥ 18 years of age, with a culture-positive clinical diagnosis of PI-associated pelvic osteomyelitis, treated by the SCI service between January 1, 2018, and October 1, 2023, was conducted. Patient characteristics, treatment choice, duration, and surgical intervention were compared between those with treatment failure versus success. Treatment failure was defined as having either microbiologic relapse, chronic suppressive therapy, or death due to the osteomyelitis during treatment. Differences between groups were assessed by Fisher’s exact test.

**Figure:**
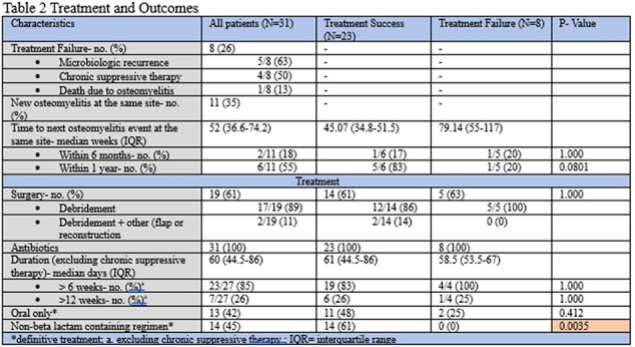

**Results:**

A total of 31 patients were identified, the majority were male (97%) with a median age of 66 years (Table 1). There were 17 patients with histopathology analyzed at diagnosis, of which 88% were positive. Majority of the cultures obtained were bone cultures (71%). The cultures were primarily polymicrobial with 45% growing MRSA (methicillin resistant *Staphylococcus aureus*) and low *Pseudomonas aeruginosa* rates (13%). Majority of patients underwent surgical debridement (61%) and received prolonged durations of antibiotics beyond 6 weeks (Table 2). A total 8 (26%) patients experienced treatment failure. Surgical management and prolonged durations of antibiotics did not differ significantly between the groups. Patients in the treatment success group had lower rates of beta-lactam as a component of their treatment regimen.

**Figure:**
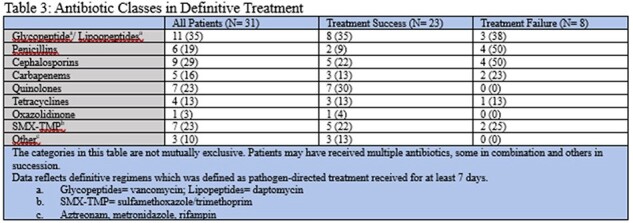

**Conclusion:**

Only a quarter of the SCI patients with PI-associated pelvic osteomyelitis failed treatment. Significantly more patients in the treatment success group had non-beta lactam containing treatment regimens. Larger studies are warranted to shed light on optimal management of pelvic osteomyelitis in this population.

**Disclosures:**

**Jaclyn A. Cusumano, PharmD, BCIDP**, Basilea Pharmaceutica: Grant/Research Support

